# Impact of a Preoperative Exercise Program on General Fitness in Patients Awaiting Bariatric Surgery: A Pilot Randomized Trial

**DOI:** 10.7759/cureus.22566

**Published:** 2022-02-24

**Authors:** Krista Hardy, Karen Kwok, Danielle R Bouchard, Neha Bharti, Dean Gamey, Ashley Vergis

**Affiliations:** 1 Surgery, University of Manitoba, Winnipeg, CAN; 2 Kinesiology, University of New Brunswick, Fredericton, CAN; 3 Kinesiology and Recreation Management, University of Manitoba, Winnipeg, CAN

**Keywords:** 6-minute walk test, preoperative exercise, physical activity, weight-loss intervention, bariatric surgery

## Abstract

Background

Evidence supports the association between exercise and outcomes following bariatric surgery. However, there is a lack of knowledge regarding the short-term benefits of preoperative exercise.

Objectives

The objective of this pilot study was to evaluate the feasibility and functional benefits of a 12-week preoperative exercise program in patients awaiting bariatric surgery. The primary aim was the six-minute walk test (6MWT). The secondary aim of this study included anthropometric measures, strength, and quality of life.

Methods

A total of 54 patients were enrolled in this pilot randomized controlled study. Of them, 29 patients received standard multidisciplinary preoperative care, while 25 patients participated in a 12-week supervised exercise program in addition to standard preoperative care consisting of strength and aerobic exercises three times per week in a fitness facility. The primary outcome was improvement in 6MWT. Secondary outcomes included other functional outcomes, quality of life, and anthropometric measures.

Results

Average attendance for the intervention group was 27.2 (75.6%) of 36 sessions. There was a mean improvement of 27 ± 10 meters in the intervention group compared with a reduction of 5 ± 10 meters in the control group (p = 0.003). Patients in the intervention group had significant improvement in all self-reported quality-of-life domains, particularly in the variables related to symptoms, hygiene, and emotions.

Conclusions

A 12-week preoperative exercise intervention was feasible and showed association with a statistically significant improvement in 6MWT and quality-of-life measures in patients awaiting bariatric surgery. The results of this study will inform sample size calculations and recruitment planning for a future study that will assess the longer-term benefits of a pre-surgical fitness intervention.

## Introduction

Obesity is a significant global burden. Its prevalence has doubled in the last 35 years, with 39% of the world’s adult population being overweight and 13% meeting the criteria for obesity [1]. Most individuals achieve a modest weight loss of 5-7% with lifestyle interventions including diet, exercise, and behavioral modification [2]. Only 20% of adults who are trying to lose weight successfully attend to calorie restriction and exercise for at least 150 minutes per week [3]. Bariatric surgery is recommended for obese patients who are unable to lose weight despite lifestyle modifications.

Bariatric surgery is the most efficacious intervention for sustained weight loss, comorbidity resolution, and mortality reduction [4]. According to the IFSO (International Federation for the Surgery of Obesity and Metabolic Disorders) Global Registry data, the roux-en-Y gastric bypass (RYGB) (38.2%) and the sleeve gastrectomy (46.0%) are the most commonly performed bariatric procedures worldwide. The RYGB is associated with 70% excess weight loss at two years following the procedure [5]. Several studies have demonstrated improved weight loss, comorbidity resolution, and mortality reduction with bariatric surgery [4].

Despite the initial success of most bariatric operations, there is a well-described incidence of weight recidivism [6]. Patients experience a mean weight regain of 12% of total body weight or 35% of maximal weight lost 10 years post-RYGB [7]. Research has focused on self-reported physical activity as well as organized fitness interventions in relation to postoperative weight loss and overall fitness. Systematic reviews report improved weight loss one year after surgery with exercise compared to those who exercise minimally (1.94-3.62 kg greater) [8-10]. Despite this, few studies have examined the effects of an organized exercise program on weight loss and fitness either before or after surgery. Postoperative exercise has been linked to improved functional capacity [9,11,12]. Three small studies of preoperative exercise have shown positive trends in aerobic capacity with moderate-vigorous physical activity [12-14]. It is unknown whether these changes are sustained postoperatively.

The objective of this study was to evaluate the feasibility and short-term functional benefits of a 12-week preoperative exercise program in patients awaiting publicly funded bariatric surgery. The primary outcome was improvement in the six-minute walk test (6MWT). Secondary outcomes included changes in anthropometry, other functional outcomes (e.g., strength), and quality of life. It was hypothesized that a preoperative exercise intervention would result in improved general fitness for patients awaiting bariatric surgery.

## Materials and methods

Overview

This was a pilot randomized trial of adult patients undergoing publicly funded bariatric surgery where patients received standard preoperative care versus the addition of a 12-week preoperative organized exercise intervention. Primary (6MWT) and secondary outcomes (anthropometric measures, strength, and quality of life) were captured at baseline and after the 12-week intervention from randomization.

Subjects

All adult patients (>18 years old) awaiting publicly funded bariatric surgery in Manitoba were introduced to the study by a certified trainer from the Canadian Society for Exercise Physiology at the Centre for Metabolic and Bariatric Surgery (CMBS), Winnipeg, Manitoba, Canada, over a 15-month period. Inclusion criteria were tentatively scheduled for bariatric surgery within six months and able to participate in an exercise program. Exclusion criteria included orthopedic, neurologic, or cardiopulmonary conditions that precluded exercise, wheelchair-bound patients, inability to tolerate moderate physical activity, and inability to commit to attending regular exercise sessions.

Eligibility for publicly funded bariatric surgery at CMBS is based on the National Institutes of Health (NIH) guidelines of body mass index (BMI) > 35 kg/m2 with obesity-related comorbidities or BMI > 40 kg/m2 and absence of untreated psychiatric or substance abuse disorders [15]. Patients referred to the CMBS are invited to attend a multidisciplinary information seminar before enrolling in the program. All patients formally enrolled in the CMBS program are evaluated by a multidisciplinary team consisting of fellowship-trained bariatric surgeons, nurse navigators, dieticians, psychologists, and kinesiologists.

Design

A total of 104 patients awaiting bariatric surgery who completed a Permission to Contact Form were contacted to participate in the study. Fifty patients declined participation in the study for various reasons after consultation with the study coordinator. Written informed consent was obtained from 54 patients at the initial appointment. Patients were assigned to the control or intervention groups after consent using a 1:1 randomization in blocks of 10 using a sealed envelope generated in SPSS Version 23 (IBM Corp., Armonk, NY, USA) by a third party at the time of consent.

Intervention

Patients in both the control and intervention groups received standard preoperative care consisting of two to four visits to the multidisciplinary team over a six-month time period. All patients attended a minimum of two visits, and additional visits were required to address vitamin or mineral deficiencies or failure to achieve program goals such as nutrition or exercise tracking. Patients were provided exercise counselling led by the Canadian Society for Exercise Physiology Certified Exercise Physiologist (CSEP-CEP) where they discussed barriers to exercise and formulated an activity plan. Patients were required to complete Craving Change™, a program that focuses on dietary behavior modification. Demonstration of behavior modification that included tracking of caloric intake and physical activity was required prior to surgery. This was submitted in either electronic or paper form by patients and reviewed at follow-up appointments. Patients failing to comply with program requirements were reviewed by the team before formal discharge from the program. All patients consumed a liquid diet consisting of 900 calories two to three weeks prior to their scheduled operation to reduce hepatomegaly and facilitate the operation.

Patients in the intervention group participated in a 12-week exercise program at a medically certified fitness center in addition to standard preoperative care. Patients completed a health screening questionnaire and underwent a graded exercise test (GXT) prior to starting the intervention to ensure that there were no cardiac or respiratory conditions that would exclude them from the study. If patients tested positive on the GXT, they were subsequently referred by the center’s medical team to a cardiologist to be cleared for exercise.

Patients in the intervention group met with a trainer for an orientation and tour of the facility. One-repetition-maximum (1-RM) testing was performed at the initial appointment for the seated row, seat chest press, leg press, and lat pulldown. This was performed to precisely measure the maximum strength for each exercise and was used to determine the initial loads to be lifted. For each exercise session, participants were asked to walk on the indoor track for 400 meters, and to do 10-25 minutes of cardiovascular exercises, six resistance training exercises, and another 10-25 minutes of cardiovascular exercises. For each exercise session, participants were given a heart rate (HR) monitor to have live feedback on their intensity. The goal was to perform 60-75 minutes of exercise three times per week after the first six weeks. For the first two weeks, participants were supervised one on one by a CSEP-CEP. In the remaining 10 weeks, they had access to facility staff just as other members of the fitness facility and could attend at their convenience. Adherence was assessed via an electronic swiping card and confirmed with exercise logbooks. When participants missed a full week of training, a call was made by a research assistant.

Resistance Training

Each participant was given a goal to lift 60% of 1-RM for 12 to 15 reps for six exercises: the seated row, the seated chest press, the leg press, lat pulldown, overhead shoulder press, and the abdominal plank. During the first week, one series was performed with the goal of reaching three series by week 4. Once three series were possible, the loads were increased by 5% for lower body exercises and 10% for upper body exercises when participants could perform more than 15 repetitions. The 1-RM evaluation was repeated at mid-intervention and loads were readjusted.

Cardiovascular Training

The participant’s goal HR was also estimated using the 220-age formula and resting HR for aerobic exercises so that each participant was reaching a minimum of 40% of HR reserve [(Max HR - Resting HR)*.40 + Resting HR] [16].

Primary outcome

The primary outcome was the 6MWT, which is the distance a patient can walk unassisted on a flat surface in six minutes. The 6MWT was performed in an unobstructed corridor where a distance of 20 meters was marked using tape in 5-meter bouts. Participants were instructed to walk along the corridor for six minutes at a self-selected pace. Patients were not encouraged during this test and were not informed how much time was remaining. This outcome was selected as it has a good correlation with peak oxygen uptake via an exercise test and is well validated in the obese population [17,18]. The 6MWT was a feasible outcome that was also inexpensive and reproducible and could be easily performed [19].

Secondary outcomes

Secondary outcomes, including anthropometric, strength, and quality-of-life measures, are defined in Table 1.

**Table 1:** Methods for secondary outcomes

Secondary outcomes	Definitions
Anthropometric measurements	Height (m), weight (kg), body mass index (BMI). Neck circumference was measured midway of the neck, below the laryngeal prominence. Waist circumference was measured at the iliac crest during the end of normal expiration while the patient was standing. Hip circumference was measured below the hips at the maximum circumference of the buttocks while the patient was standing. Each measurement was performed twice, and if there was a difference greater than 0.5 cm, a third measurement was performed.
Chair stand test	The patient began in a sitting position in a chair without arms and stood up as many times as possible within a 30-second time period. The number of total repetitions involving complete knee extension was recorded. This test is used to test leg strength and endurance.
One leg stance test	The patient removed their shoes and stood on one leg with their arms crossed. The test was stopped if the foot in the air touched the ground, the standing foot moved, the arms moved, or 45 seconds was reached. The duration of time the patient stood on one leg was recorded. This test was performed twice with the patient’s eyes open and twice with their eyes closed. This test is used to measure postural stability and balance.
Sit and reach test	The patient sat at the edge of their chair with one leg bent at 90 degrees and the other leg fully extended with their feet on the floor. The patient placed one hand on top of the other and reached forward toward their toes by bending at the hip while exhaling. The patient was required to hold this position for two seconds, and the distance from the patient’s great toe to fingertips was recorded. A negative score was recorded if the patient’s fingers did not reach beyond the toes, and a score of 0 was recorded if the fingers touched the toes. A positive score was recorded if the patient’s fingers extended beyond their toes. This test was repeated twice. This test is used to measure flexibility of the lower back and hamstrings.
Handgrip test	The patient held a handgrip dynamometer at 45 degrees from their body and squeezed their hand with maximum isometric effort for five seconds. The test was performed twice for both the dominant and non-dominant hands. Averages of both scores were recorded. This test is used to measure the maximum isometric strength of the hand and forearm and is a general test of strength.
Quality of life	The patient’s quality of life was assessed using the Laval questionnaire. This questionnaire consists of 44 questions pertaining to six domains: (1) activity/mobility, (2) symptoms, (3) personal hygiene/clothing, (4) emotions, (5) social interactions, and (6) sex life. This tool utilizes a 7-point Likert scale and a high score corresponds to a good quality of life. Average scores were reported for each domain using the rubric of corresponding questions.

Statistics

A sample size of 33 per arm was estimated in order to observe a 70-meter improvement in 6MWT (two means, two-sided t-test [alpha 0.05, power 80%]).

Baseline comparisons were made using Student’s t-test. Comparison of means at baseline and following 12-week intervention between the control and intervention groups was made with a mixed model using restricted maximum likelihood. A p-value of <0.05 was considered statistically significant. Pearson’s correlation was used to assess the relationship between % sessions attended and primary/secondary outcomes. The SPSS Statistics Program was used for all statistical analyses.

## Results

**Figure 1:**
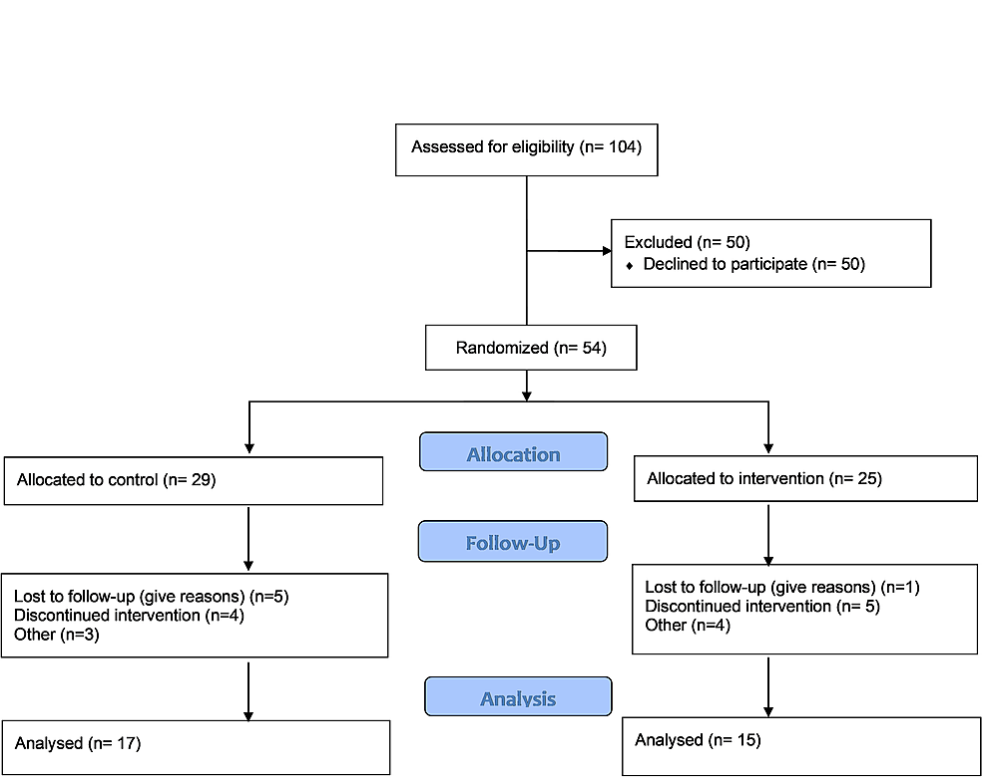
CONSORT flow diagram for patient allocation, follow-up, and analysis CONSORT, Consolidated Standards of Reporting Trials

A total of 54 patients were enrolled in the study. Of them, 29 patients were randomized to the control group and 25 patients were randomized to the intervention group. There were 22 dropouts from the study. Of the 29 patients in the control group, 12 (41.4%) dropped out, and of the 25 patients in the intervention group, 10 (40.0%) dropped out (Figure 1). All of the "dropouts” in the control group were lost to follow-up (failure to respond to telephone contact for appointments), whereas time commitment was the primary reason for dropouts from the intervention group (N=10). We undertook a completers-only analysis of the reported outcomes. There was no statistically significant difference between the gender of the patients who remained in the study or who dropped out. Patients in the intervention group were asked to attend three exercise sessions per week for 12 weeks. The average number of sessions attended by the 15 patients in the intervention group was 27.21 (75.6%) of 36 sessions. Three patients had positive GXT results that required cardiology consultation at initial assessment. All patients were subsequently cleared for exercise. No patients were removed from the program during the study for failure to meet program expectations.

Baseline characteristics

There were no significant differences in baseline characteristics between the two groups (Table 2). However, there was a higher prevalence of osteoarthritis in the control group than in the intervention group (p = 0.01).

**Table 2:** Prevalence of comorbidities in the control and intervention groups *Treated with continuous positive airway pressure or documented on sleep study BMI, body mass index; OSA, obstructive sleep apnea

Variable	Control group	Intervention group	p-Value
Age (years)	46.7	47.5	0.44
BMI (kg/m^2^)	45.2	46.3	0.79
Females (%)	63.0	88.0	0.08
Diabetes (%)	22.7	45.0	0.23
Hypertension (%)	45.5	55.0	0.76
Hyperlipidemia (%)	18.2	20.0	0.88
Cardiovascular disease (%)	4.5	0.0	0.33
OSA* (%)	36.4	35.0	0.93
Osteoarthritis (%)	45.5	5.0	0.01

6MWT

The intervention group experienced a statistically significant increase in 6MWT distance by 27.46 ± 10 meters, while the control group reduced their distance by an average of 5.00 ± 10 meters. When comparing the change in 6MWT between completers in the control and intervention groups, the difference was 32.34 ± 14.29 meters favoring the intervention group (p = 0.03) (Table 3).

**Table 3:** Pre- and post-6MWT results Values are presented as mean (SD) or as change (SE)

	Baseline (meter)	p-Value	Delta (meter)	p-Value	∆Between groups (meter)	p-Value
Control (N=27)	473.03 (97.95)	0.80	-4.88 (10.01)	0.63	32.24 (14.29)	0.03
Intervention (N=25)	460.10 (50.92)		27.46 (10.34)	0.01		

Secondary outcomes: anthropometric variables

After 12 weeks, there was no significant change in BMI or hip circumference in either group. However, both groups experienced a significant reduction in neck and waist circumference (Table 4).

**Table 4:** Pre- and post-anthropometric measures Control: n=27; Intervention: n=25 Values are presented as mean (SD) or as change (SE) BMI, body mass index

Variable		Baseline	p-Value	Change from baseline	p-Value	Intervention vs. control change	p-Value
BMI (kg/m^2^)	Control	45.73 (5.34)	0.44	-0.40 (0.42)	0.35	-0.17 (0.62)	0.78
	Intervention	46.89 (5.40)		-0.57 (0.45)	0.21		
Neck circumference (cm)	Control	43.89 (4.33)	0.30	-1.05 (0.39)	0.01	-0.19 (0.57)	0.74
	Intervention	42.54 (4.82)		-1.24 (0.41)	0.01		
Waist circumference (cm)	Control	134.14 (13.11)	0.50	-3.04 (1.04)	0.01	-2.40 (1.52)	0.13
	Intervention	131.83 (11.42)		-5.45 (1.11)	0.00		
Hip circumference (cm)	Control	135.60 (22.34)	0.16	2.54 (4.71)	0.59	-11.67 (6.87)	0.10
	Intervention	143.12 (14.20)		-9.13 (4.99)	0.08		

Secondary outcomes: other functional measures

A significant increase in flexibility was observed in the control group and a significant increase in chair stand repetitions was demonstrated for the intervention group (Table 5). There was no difference in functional outcomes between groups.

**Table 5:** Comparison of strength measures between control and intervention groups at baseline and following 12-week intervention Control: n=27; Intervention: n=25 Values are presented as mean (SD) or as change (SE)

Variable		Baseline	p-Value	Change from baseline	p-Value	Intervention vs. control change	p-Value
Chair stand repetitions	Control	11.85 (4.09)	0.19	1.19 (0.78)	0.13	1.14 (1.11)	0.31
	Intervention	13.12 (2.47)		2.33 (0.80)	0.01		
Flexibility	Control	-0.59 (13.73)	0.31	5.65 (2.69)	0.04	-3.28 (3.93)	0.41
	Intervention	2.64 (7.80)		2.38 (2.86)	0.41		
Balance (eyes open)	Control	21.27 (16.17)	0.77	-0.18 (3.15)	0.96	3.01 (4.59)	0.52
	Intervention	22.51 (14.54)		2.83 (3.34)	0.40		
Balance (eyes closed)	Control	4.75 (4.76)	0.76	0.30 (0.86)	0.73	-0.84 (1.26)	0.51
	Intervention	5.22 (6.15)		-0.54 (0.92)	0.56		
Handgrip strength (dominant hand)	Control	36.82 (15.56)	0.23	0.74 (1.24)	0.55	0.98 (1.81)	0.59
	Intervention	32.18 (11.70)		1.72 (1.32)	0.20		
Handgrip strength (non-dominant hand)	Control	31.87 (15.09)	0.48	0.79 (1.32)	0.56	0.86 (1.93)	0.66
	Intervention	29.24 (11.07)		1.65 (1.41)	0.25		

Secondary outcomes: quality of life

At baseline, patients in the intervention group had better scores for activity and symptoms compared to the control group (Table 6), corresponding to a better quality of life. After the 12-week intervention, there were no response changes in patients in the control group, whereas there were statistically significant improvements in all domains for the intervention group. When comparing the differences between the intervention and control groups, there were statistically significant changes for the categories of symptoms, hygiene, and emotions (Table 6).

**Table 6:** Comparison of Laval questionnaire scores between the control and intervention groups at baseline and following 12-week intervention Control: n=26; Intervention: n=25

Variable		Baseline	p-value	Change from baseline	p-value	Intervention vs. control change	p-value
Activity	Control	4.38 (1.29)	0.02	0.43 (0.22)	0.06	0.30 (0.32)	0.35
	Intervention	5.20 (1.01)		0.73 (0.23)	0.00		
Symptoms	Control	4.49 (1.02)	0.05	0.003 (0.15)	0.98	0.67 (0.23)	0.01
	Intervention	4.96 (0.63)		0.68 (0.16)	0.00		
Hygiene	Control	4.90 (1.34)	0.19	-0.28 (0.17)	0.11	0.93 (0.25)	0.00
	Intervention	5.32 (0.84)		0.65 (0.18)	0.00		
Emotions	Control	3.93 (1.40)	0.39	0.31 (0.18)	0.10	0.55 (0.26)	0.05
	Intervention	4.23 (1.09)		0.85 (0.19)	0.00		
Social	Control	4.71 (1.47)	0.69	0.40 (0.20)	0.05	0.42 (0.29)	0.16
	Intervention	4.87 (1.31)		0.82 (0.21)	0.00		
Sex	Control	4.10 (1.83)	0.44	0.09 (0.28)	0.75	0.54 (0.41)	0.19
	Intervention	4.40 (1.32)		0.63 (0.30)	0.04		

## Discussion

This study demonstrated that a preoperative exercise intervention was feasible and associated with a modest improvement in 6MWT as well as quality-of-life measures in patients awaiting bariatric surgery.

In the current literature, a few studies have examined the effects of structured exercise programs around the time of bariatric surgery. Stegen et al. enrolled 15 morbidly obese patients who self-selected into exercise or control groups after surgery [9]. They evaluated the patient’s BMI, functional capacity, and aerobic capacity prior to surgery and at four months postoperatively. Eight patients in the intervention group exercised three times per week for 12 weeks starting one month after their surgery. Those in the exercise group had an improvement in aerobic capacity and functional capacity. A randomized controlled trial performed by Castello et al. assigned 21 patients to training and 31 to control [11]. The intervention group participated in a treadmill-based exercise program three times per week for 12 weeks postoperatively. The study concluded that 12 weeks of aerobic training improves cardiac autonomic modulation and functional capacity four months after bariatric surgery [12].

Only a few studies have examined preoperative exercise programs in patients awaiting bariatric surgery [12-14]. A cohort study of 12 participants enrolled in a 12-week preoperative exercise program consisting of three endurance and strength training sessions per week for 12 weeks [13] found improved weight, physical fitness, and quality of life post-intervention. They concluded that a preoperative exercise program was a feasible intervention in patients awaiting bariatric surgery. An unblinded pilot trial randomized 22 patients into a gym-based exercise program (n=7), home-based exercise program (n=8), and control (n=7) for eight weeks [14]. Patients in these exercise programs performed 30-minute aerobic training sessions and two resistance exercise sessions three times per week. There was a trend toward improved aerobic capacity and preoperative weight loss in the gym-based group, but the results were not statistically significant. The largest study, the Bari-Active study [12], enrolled 75 participants who were randomized to a six-week physical activity intervention involving weekly sessions with instructions in behavioral strategies versus standard care. Patients enrolled in the intervention group had increased moderate-to-vigorous physical activity preoperatively.

Patients in our intervention group showed a statistically significant improvement in 6MWT distance when compared to patients in the control group. In the current literature, there are no established reference values for 6MWT in obese patients and particularly for bariatric surgery patients. The distance walked is correlated to age, gender, and BMI [20]. Baseline 6MWT results were 473.03 meters in the control group and 460.10 meters in the intervention group. This is lower than the values found in studies by Capodaglio et al. of 563.60 meters [20] or Hulens et al. of 538.9 meters [21]. This difference could be accounted for by the fact that our patients were older (control mean age of 46.69 years and intervention mean age of 47.53 years vs. mean age of 35.93 years in Capodaglio et al.’s study [20] vs. mean age of 38.9 years in Hulens et al.’s study [21]) as well as more obese (control mean BMI of 45.21 kg/m2 and intervention mean BMI of 46.27 kg/m2 vs. mean BMI of 43.39 kg/m2 in Capodaglio et al.’s study [20] vs. mean BMI of 40.7 kg/m2 in Hulens et al.’s study [21]).

The modest improvement in 6MWT needs to be interpreted within the clinical context as well. A minimum clinically important difference (MCID) reflects the change that occurs from a clinical intervention that is meaningful to the patient [22]. This reflects the concept that there may be statistically significant changes secondary to an intervention, but there may not be clinical significance [23]. A statistically significant increase in 6MWT is often less than a clinically significant increase in an individual [24]. There is debate as to whether an MCID exists for 6MWT [25,26], and there is limited literature examining MCID for 6MWT in obese patients.

Larsson and Reynisdottir found good reproducibility and group validity for 6MWT in obese patients and concluded that an improvement of at least 80 meters or 15% was required to make the difference clinically significant [27]. However, this was not in the setting of patients undergoing bariatric surgery. The patients in our intervention group showed a statistically significant increase in a 6MWT distance of +27.46 ± 10.34 meters (p = 0.01) or a 6% improvement. While the clinical significance of this value is still undetermined, it is a positive result, and further research is warranted to examine the durability of 6MWT results postoperatively. The sustainability of this change postoperatively will be of clinical significance. Our sample size calculations were based on Larsson’s data and were underpowered to detect 80 meters due to recruitment/time limitations in our study. A larger study could potentially detect this level of change.

Secondary outcomes

There were positive trends for the intervention group but no statistically significant changes in BMI or in neck, hip, or waist circumference when compared to the control group. The CMBS program does not focus on weight loss preoperatively, and there is no weight loss requirement to be approved for surgery.

There were no statistically significant differences in the chair stand test [28], flexibility [29], balance, and handgrip strength [30] when comparing the control and intervention group. Our exercise prescription followed the American College of Sports Medicine (ACSM) and Centers for Disease Control and Prevention (CDC) guidelines [31] and included resistance training in order to improve muscle strength and endurance. Typical resistance training programs include 8 to 12 repetitions for 8 to 10 separate exercises [32]. However, baseline data have demonstrated that our bariatric patients spend less than 6% of time in moderately vigorous physical activity, and therefore realistic goals need to be set in a 12-week program [33]. It is possible that strength benefits might be seen with a more longitudinal intervention.

Patients in the intervention group had higher scores for activity and symptoms than those in the control group, which corresponds to a better quality of life. Surveys are completed prior to randomization; therefore, it is unlikely that group allocation influenced the patient responses.

The intervention group showed improved quality of life in all categories after completing the intervention. There were statistically significant changes for the symptoms, hygiene, and emotion categories compared to the control group. Therrien et al. studied the Laval questionnaire and attempted to determine the MCID for each domain [34]. The change in scores for the intervention group exceeded the MCID for activity (0.69) and symptoms (0.64) but did not meet the criteria for hygiene (1.21), emotions (1.0), social interactions (0.97), and sexual life (1.91) [34]. This improved perception of well-being is very important and could be used to motivate patients to change their lifestyle and sustain these changes following bariatric surgery.

There is no association between the percentage of exercise sessions attended and change in primary or secondary outcome measures in the intervention group. However, other factors to be considered include whether targets were met at each session, the patient’s exercise intensity, and whether patients increased their activities appropriately. The exercise logbooks were used to monitor progress, and the fitness consultants provided feedback to encourage appropriate exercise.

Limitations

This study demonstrated that short-term implementation of preoperative exercise can increase 6MWT. This is a significant finding as 6MWT is a well-established measure of overall fitness. The lack of improvement in secondary outcomes might relate to the duration of the intervention, the overall attendance/participation, the specific design of the aerobic and resistance exercises in the program, or the lack of power to detect differences in these outcomes.

Patients are usually prepared for bariatric surgery over a few months, and therefore it would be difficult to extend a preoperative intervention beyond 12 weeks. Attempts were made to follow up on patients who did not attend sessions to optimize participation. The program was also designed based on patient baseline fitness and strength with targeted outcomes in line with standard kinesiology interventions.

This pilot study was not designed to assess the longer-term postoperative benefits of pre-surgical fitness intervention. In the future, a study on the longer-term benefit of pre-surgical fitness is needed in order to develop guidelines to incorporate an exercise program before bariatric surgery.

Studies using exercise interventions commonly report dropout rates of 25-50% [35,36]. This is often due to patients being lost to follow-up or due to the time commitments of the study. Future study will require a more significant dropout rate in the sample size calculation.

Patients approved for bariatric surgery have several appointments with the multidisciplinary team at the CMBS. Follow-up could potentially be improved by scheduling study appointments in conjunction with other mandatory appointments in order to reduce time commitments. Patients were required to attend all sessions at the fitness facility and were not provided a home option. It is possible to consider a combined program with home and facility options. However, there are limitations to home exercise programs, including the type of exercise conducted, documentation of participation, and patient motivation.

Ongoing and further research

The short-term benefits of exercise are demonstrated in several small preoperative studies including the current study. The question of sustained improvement in fitness and associated weight loss needs to be addressed with more longitudinal studies. Further research will examine factors such as the duration of intervention, timing of intervention (pre- vs. postoperatively or both) as well as types of activities performed during the intervention, and a behavioral modification component to facilitate adherence. Additionally, future directions of the study will explore whether exercise routines implemented prior to undergoing bariatric surgery were maintained postoperatively.

## Conclusions

This preoperative exercise intervention was feasible and was associated with a significant improvement in 6MWT in patients awaiting bariatric surgery. There were also significant changes in quality-of-life domains, particularly for the categories of symptoms, hygiene, and emotions in the intervention group. This perceived improvement in quality of life is important and should be used to motivate patients to change their lifestyle prior to and after bariatric surgery. Future research should examine if a preoperative exercise program will impact fitness outcomes after bariatric surgery. It should also explore whether patients maintained the exercise routines postoperatively.
